# *In Silico* Tools for Analysis of Single-Nucleotide Polymorphisms in the Bovine Transferrin Gene

**DOI:** 10.3390/ani12060693

**Published:** 2022-03-10

**Authors:** Aarif Ali, Muneeb U. Rehman, Syed Mudasir Ahmad, Tabish Mehraj, Ishraq Hussain, Ahmed Nadeem, Manzoor Ur Rahman Mir, Showkat Ahmad Ganie

**Affiliations:** 1 Department of Clinical Biochemistry, School of Biological Sciences, University of Kashmir, Hazratbal, Srinagar 190006, J&K, India; buttaarif31@gmail.com; 2 Division of Veterinary Biochemistry, Faculty of Veterinary Sciences & Animal Husbandry, SKUAST-Kashmir, Shuhama Campus (Alusteng), Ganderbal 190006, J&K, India; ishraq@skuastkashmir.ac.in (I.H.); manzoorvbc43@gmail.com (M.U.R.M.); 3 Department of Clinical Pharmacy, College of Pharmacy, King Saud University, Riyadh 11451, Saudi Arabia; 4 Division of Animal Biotechnology, Faculty of Veterinary Sciences & Animal Husbandry, SKUAST-Kashmir, Shuhama Campus (Alusteng), Ganderbal 190006, J&K, India; mudasirbio@skuastkashmir.ac.in; 5 Department of Pharmaceutics and Drug Delivery, School of Pharmacy, University of Mississippi, Oxford, MS 38677, USA; tmehraj@go.olemiss.edu; 6 Department of Pharmacology & Toxicology, College of Pharmacy, King Saud University, Riyadh 11451, Saudi Arabia; anadeem@ksu.edu.sa

**Keywords:** subclinical mastitis, transferrin, SNP, sequencing, in silico tools

## Abstract

**Simple Summary:**

Subclinical mastitis represents a significant disease in dairy animals and negatively impacts udder health. Genetics, pathogens, and poor health and management procedures in dairy animals contribute primarily to this disease. Animal genetics plays a major role in disease resistance and susceptibility. Studying single-nucleotide polymorphisms in genes helps to detect resistant and susceptible animals. Transferrin is one of the important glycoproteins involved in the innate immune system that prevents iron availability to pathogens. Several studies have reported polymorphism in the transferrin gene in dairy animals with mastitis, but no detailed information on in silico tools is available for identification and verification. So, this study was carried out to detect polymorphism in the transferrin gene in healthy and mastitic cows, and to use various in silico based tools and software to find the impact of single-nucleotide polymorphisms (SNPs) on animal health. The results indicated three SNPs were located in the transferrin gene of Holstein Friesian cross-bred dairy cows. Moreover, the use of online computational tools provided significant knowledge regarding whether the SNPs would be deleterious, damaging, benign, neutral, or affect protein stability and function.

**Abstract:**

Dairy cattle with a high milk yield are susceptible to many infectious diseases, such as mastitis. Subclinical mastitis (SCM) is the most prevalent form of mastitis that predominantly affects animal health, and causes adverse effects on the quality and quantity of milk. In dairy animals, subclinical mastitis often remains undetected, as no gross changes in udder characteristics are visible. In the present study, 135 Holstein Friesian dairy animals were selected and screened as healthy (*n* = 25) and mastitic (*n* = 110) based on diagnostic tests such as the California mastitis test, pH, electrical conductivity, and somatic cell count. In this study, the somatic cell count was used as a gold-standard test in differentiating subclinical mastitis animals from healthy ones. The present study was carried out to study polymorphisms in the bovine transferrin gene in cows (with subclinical mastitis and healthy). For the early detection of resistant/or susceptible animals, a useful marker could be provided by the detection of single-nucleotide polymorphisms (SNPs) in the transferrin gene, which are often associated with mammary innate immune response. The sequencing results revealed three nucleotide substitutions: two transversions (230 A > C, 231 C > A) and one transition (294 A > G) in susceptible cows as compared to disease-free subjects. The nucleotide variations at position 230 (GAC > GCA) and 231 (GAC > GCA) were nonsynonymous, and corresponded to an amino acid change from aspartic acid to alanine; whereas at position 294 (GAA > GAG), the mutation was synonymous. In the present study, many in silico tools were taken into consideration to determine the effect of SNPs on protein structure and function. The PROVEAN tool found the amino acid substitution to be neutral and deleterious. PolyPhen-2 revealed the amino acid variations at positions 320 and 321 to most likely be damaging; and at the 341 position, the variations were benign. The I-Mutant and MUpro tools found that the protein stability decreased for nonsynonymous variations. The SIFT tool revealed the protein function was likely to be affected in nonsynonymous variations, with no change in the case of synonymous ones. Phylogenetic analysis of the bovine transferrin gene revealed a close relation of the CA allele with the *Bos taurus* transferrin, while the G allele was closely related to a cross of *Bos indicus* × *Bos taurus* serotransferrins, followed by the *Bison bison* transferrin. The least relation was shown by both alleles to *Capra hircus*, *Ovis aries*, and *Bubalus bubalis*.

## 1. Introduction

The ability to predict a phenotype for disease resistance is quite difficult in dairy animals. It is quite unlikely that some healthy animals are not sufficiently exposed to the diseased organism to become infected. Apparently, healthy-looking animals might have subclinical infections and act as pathogen reservoirs. However, in disease-resistant animals, a major hurdle is the proper selection and identification of exact phenotypes in order to develop efficient genetic markers with good prognostic values. In molecular genetics, recent technologies for analysis of genomes have led to detection of gene markers that are associated with economic traits. Thus, marker-assisted selection that optimizes effective, rapid, and accurate genetic development is highly required. In candidate genes, it is important to study genetic variations, as well as their associations with somatic cell count (SCC) and milk yield [1,2]. In the last few years, various studies have stressed the identification and characterization of markers associated with milk yield and fat traits [3,4]. In addition to quantitative trait locus (QTLs), several genetic polymorphisms have been identified in genomes, gene regions, or genetic markers related to disease resistance and susceptibility. The simplest type of polymorphism is the single-nucleotide polymorphism (SNP). In a population, a variation in the nucleotide sequence that is observed at a frequency of 1% or more is referred to as an SNP, and these differences provide diversity among populations [5]. The diversity of nucleotide bases is four times lower within the coding exons, with around half resulting in nonsynonymous variations in codons. The SNPs located in the coding regions are most likely to cause functional changes. In genomes, nucleotide bases are of two types: purines (adenine and guanine) and pyrimidines (cytosine and thymine). Similarly, the nucleotide variations are of two types: transition and transversion. In transition, a purine base changes to another purine nucleotide and a pyrimidine nucleotide mutates into another pyrimidine entity. Likewise, in the case of transversion, a purine moiety changes into a pyrimidine nucleotide or vice versa. The SNP could prove to be a useful tool in the detection of resistant and susceptible animals [6,7]. In addition to this, some SNPs may have vital biological, structural, and functional properties [8].

The ability of the mammary gland to combat a wide range of pathogens to produce a strong immune response, even upon the first exposure, and to engage in non-pathogen-specific methods of recognition is provided by the innate immune system. One of the genes that is a part of innate immune system, which provides defense against bacterial and fungal pathogens, is the transferrin (Tf) gene, which prevents microbial access to iron, which they require for their action in host body [9]. Tf is present in chromosome 1q41-q46, with 17 exons and 39 kb of genomic DNA, and its expression mainly increases during infection [10]. The transferrin gene encodes a β-globulin plasma protein that binds iron and is primarily produced by the liver. The Tf protein has a molecular weight of 80 kD. In transferrin, there are two iron-binding sites, each separate and capable of binding one atom of a ferric molecule. During mastitis, as the tissue permeability is increased, transferrin passes from blood to milk; henceforth, the total iron binding capacity of milk is increased. Transferrin is known to prevent adhesion of bacteria [11], in addition to having an antifungal effect that is iron-independent [12]. Transferrin limits access to microbial iron, and thus contributes to innate immune defense against bacterial and fungal pathogens [9]. The association of transferrin genotypic classes with milk fat and production have been reported, and are possibly due to Tf lipolytic activity [13,14,15]. Animals previously diagnosed with mastitis had higher concentration of transferrin in milk as compared to healthy animals. These results suggest a possible relationship between mastitis and the Tf gene in dairy cattle.

Single-nucleotide polymorphisms have been found in the Tf gene; however, due to limited availability of data on SNPs and their association with infectious diseases, particularly mastitis, they need to be examined further [16,17]. SNPs also need proper validation regarding whether they have any association with bovine mastitis resistance/tolerance. In bovine animals, many polymorphisms in the transferrin gene have been reported [16,17]. This study aimed to determine whether SNPs are a disease marker that could be useful in identifying genes and might contribute to disease susceptibility. In the present study, many online computational tools (BLAST, NovelSNPer, PROVEAN, PolyPhen-2, I-Mutant, MUpro, and SIFT) were used to assess the effects of these variations on the function and structure of SNPs [18,19]. BLAST identifies regions of common similarity between the nucleotide sequences. This program calculates the statistical significance by comparing the query nucleotide or protein sequences to sequence databases. NovelSNPer identifies whether a variation is a known variant or a previously discovered variation in the first stage. Each variation is then categorized into one of 15 classes of SNPs, or in second stage into 19 InDel classes. It can analyze two million SNPs in six hours.

Homology-based sequence tools such as the Protein Variation Effect Analyzer (PROVEAN) and Sorting Intolerant from Tolerant (SIFT) compute conservation scores to determine the deleterious properties of mutations [20,21]. PolyPhen-2 is a sequence-based structural tool that computes functional pathogenicities of nonsynonymous polymorphisms. I-Mutant is used to determine protein stability. MUpro is also a web-based algorithm that calculates free-energy changes in wild and mutated protein forms.

The primary goal of a phylogenetic analysis is to study the evolutionary history of an organism and to find its similarity with other living organisms from an evolutionary point of view. Molecular phylogenetics helps us to gather insights about biological diversity, genetic organization, and the developmental events during evolution. The phylogenetic tree comprises aligned sequences (DNA or RNA strings) [22]. The most commonly used approaches are most likely the alignment-based or aligned free methodologies [23,24,25]. In the era of computational bioinformatics, the field of molecular phylogenetics is a fundamental branch.

Therefore, in this study, an effort was made to identify SNPs in the bovine transferrin gene by using various in silico tools for analysis.

## 2. Materials and Methods

### 2.1. Study Design

The present study involved a total of 135 Holstein Friesian (HF) (110 with subclinical mastitis and 25 healthy) dairy cows that were randomly selected during the period of June 2017 to January 2019. During this period, cross-sectional studies and laboratory examinations of both milk and blood samples were undertaken. All the selected dairy cows were examined directly at the quarter level for clinical assessment of manifestations, and several screening tests (California mastitis test, pH, electrical conductivity and somatic cell count) were performed on spot for detection of subclinical mastitis. The milk samples were analyzed by using these diagnostic tests, and the animals were classified either as healthy (negative) or subclinical (positive) based on the results. Somatic cell count (SCC) is considered to be a gold-standard test for detecting SCM. The animals that were selected for this study were subjected to a detailed history, including management history, scientific procedures adopted, and parity, and other parameters such as flooring, type of feed, type of milking, udder washing, and feeding after milking were studied. From these selected areas, a variable number of apparently healthy cows (Normal) and cows with clinical signs (SCM) animals were selected after screening for the disease. The selection and grouping of animals for sampling was predominantly based on physical examinations of milk and udders, as well as screening tests for subclinical mastitis.

### 2.2. Screening Tests

In this study, screening of the dairy cows was done to identify animals with subclinical mastitis from the apparently healthy animals. The following tests were performed for animal screening.

#### 2.2.1. California Mastitis Test (CMT)

The California Mastitis Test is a simple, rapid, and easily available diagnostic test that provides a measure of the somatic cell count in milk. In this test, milk samples from the selected animals are taken in a paddle and mixed with an equimolar volume of CMT reagent (3% sodium lauryl sulphate). The basis for this test is that the CMT reagent disrupts the cell membrane of any cells present in the milk, allowing the DNA in those cells to react and precipitate, forming a gel. The intensity of gel formation determines the severity of infection. Generally, CMT scores are read as negative (0), weak positive (+1), distinct positive (+2), and strong positive (+3).

#### 2.2.2. pH

In milk samples, pH was measured using a portable digital pH meter (Eutech Singapore). The animals with a milk pH greater than 6.7 were grouped under subclinical mastitis, and those with a value lower than this were identified as healthy.

#### 2.2.3. Electrical Conductivity (EC)

A portable digital EC meter (Eutech Singapore) was used for determining milk conductivity. The animals with a milk EC of greater than 4.44 mS/cm were grouped under subclinical mastitis, and those with a value lower than this were identified as healthy.

#### 2.2.4. Somatic Cell Count (SCC)

This diagnostic test is considered to be a gold-standard test for detecting subclinical mastitis. In milk samples, SCC was measured by a portable DeLaval cell counter (DCC; DeLaval International AB, Tumba, Sweden). An SCC of >200,000 was used as a cutoff point for differentiating animals with subclinical mastitis from healthy ones.

### 2.3. Collection of Samples

Blood samples were withdrawn from the same animals from which milk was collected without harming them. From the jugular vein, 10 mL of aseptic blood was withdrawn from each animal and collected in an anticoagulant (EDTA K_3_) tube (GONG DONG^TM^—China) for DNA extraction.

### 2.4. DNA Extraction

The extraction of genomic DNA from whole blood samples was carried out using the phenol chloroform method, as given by Sambrook et al. 2001 [26]. The quality of the isolated genomic DNA was evaluated by agarose gel electrophoresis with a gel concentration of 0.8%. The purity of isolated genomic DNA was assessed with a UV spectrophotometer (Shimadzu UV-1900). In a cuvette, 5 µL of DNA was dissolved in 495 µL of TAE buffer, and the following formula was used to determine DNA quantity:

Quantity of DNA (µg/mL) = (A260 × 50 × dilution factor)

The ratio of optical density (OD) values at 260 nm to 280 nm was used as criteria for determining purity of genomic DNA.

### 2.5. PCR Amplification

A portion (14,432–14,764) of bovine transferrin gene was amplified from genomic DNA using specific primers synthesized by Integrated DNA Technologies (IDT), Bangalore, India. The primers for bovine transferrin were designed using PRIMER 3 Plus software. To check the PCR efficiency of the primers, different dilutions of DNA were used, and a calibration curve was obtained. The details of the primers are given in Table 1.

All the components required for setting of a PCR were used on ice for amplification of the bovine transferrin gene in a final volume of 25 µL (Table 2).

In the process of optimization of the PCR to obtain the desired amplified product, a range of different annealing temperatures, denaturation times, and numbers of cycles were tried initially. The conditions that provided the best results were chosen as the standard reaction conditions (Table 3).

In order to determine the size of the amplified PCR products, horizontal submarine gel electrophoresis using 2.0% agarose gel containing EtBr (5 µL/100 mL) was run continuously for 1 h at a constant voltage of 100 V using TAE as a running buffer. The size of the PCR products was evaluated by comparison with a reference molecular marker.

### 2.6. Sequence Analysis

The PCR products that yielded good results in both quality and quantity were selected and sent for DNA sequencing. The purified PCR products of the transferrin gene were commercially sequenced by Sanger’s enzymatic DNA sequencing technique at Bionivid Technology Pvt Limited (Bangalore, India) after following proper transportation instructions. All the precautions were taken into consideration before outsourcing the PCR products in order to avoid any kind of degradation. The generated sequence data of the outsourced PCR products from the Bionivid laboratory were available within two weeks, along with their agarose gel electrophoresis images for confirmation of products.

### 2.7. Bioinformatical Tools for SNP Analysis

The sequence data of the PCR products were available in Chromas format. The sequence data were analyzed by using the bioinformatical software Bio-edit v 7.2.6, BLASTn, BLASTx, and NovelSNPer. Sequence alignment of the selected DNA regions of the transferrin gene with multiple online available *Bos taurus* sequences (www.ncbi.com, last accessed on 16 April 2021) was performed using CLUSTALW. Validation of the SNPs was carried out using different online bioinformatical tools (PROVEAN, PolyPhen-2, I-Mutant, MUpro, and SIFT).

### 2.8. Identification Tools for SNP

Many tools were used for the detection of SNPs, and some of them are listed below.

#### 2.8.1. Bio-Edit

The sequencing data was received in ABI chromatogram/FASTA format, and was then analyzed. Before analysis, trimming of data was conducted to remove the initial 10–20 nucleotides and noise. The peaks in the chromatogram were properly cross-checked with the bases mentioned.

#### 2.8.2. BLAST

BLAST was used in nucleotide sequences to determine evolutionary and functional relationships, as well as to identify gene family members

#### 2.8.3. Nucleotide BLAST (BLASTn)

In this program, our user-specific DNA search queries returned the most similar DNA sequences from the database.

#### 2.8.4. BLASTx

BLASTx is a novel tool to detect mutations. This tool compares the nucleotide query sequence translational products of both strands against a database protein sequence. This online tool produces accurate and very reliable data results when dealing mostly with coding DNA. It directly helped us to see the function of a protein sequence by translating a sequence of interest prior to searching, which often provided us with more annotated protein hits.

#### 2.8.5. NovelSNPer

NovelSNPer is a software program for processing output lists quickly and efficiently. NovelSNPer is based on gene structural data provided in Ensembl.

### 2.9. Validation/Verification Tools for SNP

After the identification of SNPs, it was important to correctly validate the variations, and for this purpose, other in silico tools were used.

#### 2.9.1. PROVEAN Scores

The Protein Variation Effect Analyzer is online in silico software that determines the effects of amino acid substitutions.

#### 2.9.2. PolyPhen-2

PolyPhen-2 is an online bioinformatics database that predicts impacts on the function and structure of a protein caused by substitution of an amino acid.

#### 2.9.3. I-Mutant

I-Mutant is also an online web-based bioinformatical tool used to predict the stability of a protein.

#### 2.9.4. MUpro

MUpro is another online bioinformatical tool that predicts the stability of a protein.

#### 2.9.5. Sorting Intolerant from Tolerant (SIFT)

This algorithm is one of the standard tools used to predict variants and characterize missense variations. This tool determines the effect of coding variants (amino acids) on protein function. Depending on sequence homology and the amino acid’s physical properties, SIFT predicts whether a protein structure could be affected by an amino acid substitution.

### 2.10. Phylogenetic Analysis

In this study, MEGA X (version 2020) was used for the phylogenetic analysis. A phylogenetic analysis of the bovine transferrin gene sequences was performed with the other top 10 related transferrin sequences identified in *Bos mutus* (wild yak), *Bos grunniens* (domestic yak), *Bison bison* (buffalo), *Bos indicus* (cow), *Bubalus bubalis* (water buffalo), *Ovis aries* (sheep)and *Capra hircus* (goat).

### 2.11. NCBI GenBank

The nucleotide sequences with SNPs were submitted to the NCBI GenBank.

### 2.12. Statistical Analysis

The data obtained in the current study were analyzed using the computer-aided statistical software package GraphPad Prism (version 8). The differences between means were analyzed using an unpaired Student′s t-test, and significance was determined at *p* < 0.05.

## 3. Results

In the present study, pH, EC, and SCC values in milk samples greater than 6.7, 4.44, mS/cm and 200,000 cells/mL, respectively, were used as cutoff points for determining subclinical mastitis. In this study, pH (<0.0001), EC (<0.0001), and SCC (*p* < 0.002) were found to be statistically significant.

This study investigated single-nucleotide polymorphisms (SNPs) in the bovine transferrin gene in the local cattle population and whether they were associated with either tolerance of or susceptibility to subclinical mastitis. During this study, the data that were obtained through sequencing were analyzed with various bioinformatical tools wherever applicable.

**Figure 1:**
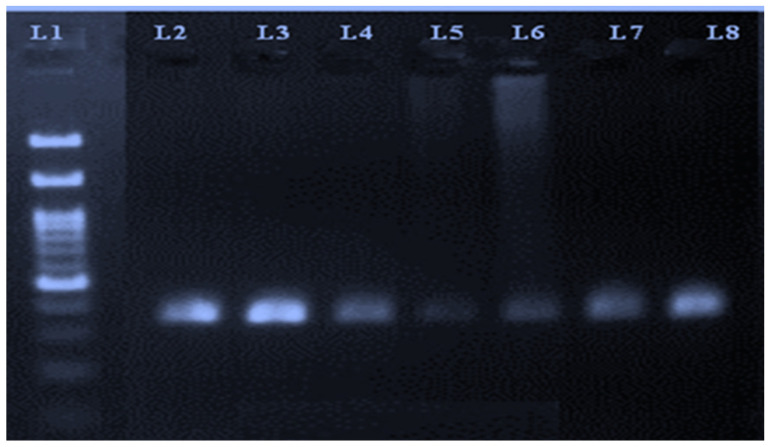
PCR amplicons of reference gene (transferrin) run on 2% agarose gel. Lane 1 represents a 100 bp ladder, whereas Lanes 2 to 8 correspond to PCR amplicon products.

A representative image of the PCR amplicons is shown in Figure 1.

### 3.1. DNA Sequencing

After outsourcing, the results for PCR-sequenced products were obtained in ABI chromatogram/FASTA format; necessary measures for data analysis were taken before outsourcing.

### 3.2. Identification Tools for Single-Nucleotide Polymorphisms

When subjected to a sequencing analysis, the 332 bp fragment of the bovine transferrin gene was found to be polymorphic when compared with the sequence of *Bos taurus* species (GenBank: Acc. no. NM_177484.3).

#### 3.2.1. Nucleotide BLAST (BLASTn)

The processing of the input nucleotide data returned sequences that produced significant alignments (Table 4).

The sequencing results revealed three nucleotide substitutions—two transversions and one transition—in subclinical mastitis cows as compared to disease-free cows (230: A > C, 231: C > A, and 294: A > G (Table 5)).

#### 3.2.2. NovelSNPer

The results of variation shown by NovelSNPer with respect to the types of SNPs and amino acid changes are given in Table 6.

#### 3.2.3. BLASTx

**Figure 2:**
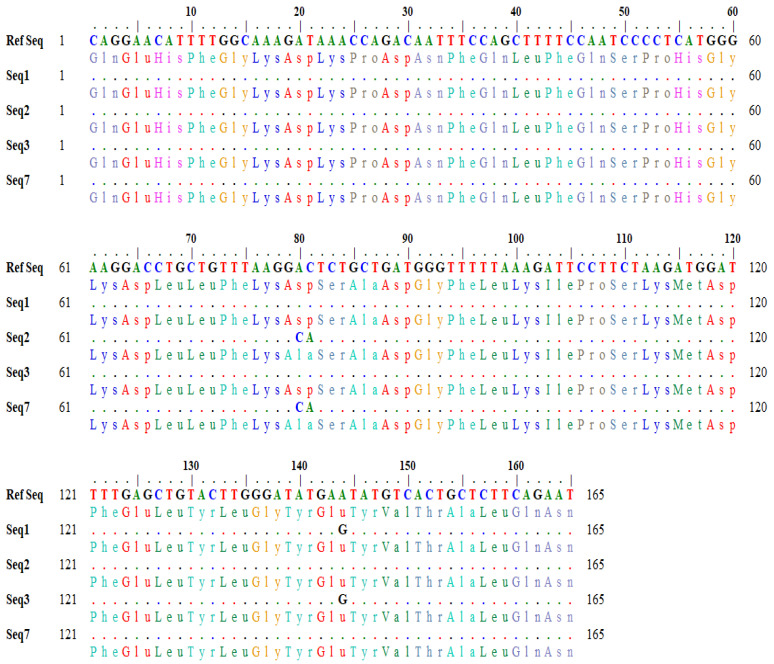
Detailed output of codons showing variations.

The detailed output of codon variation generated by the BLASTx tool is shown in Figure 2. In sequences (seq2 and seq3), the nucleotide bases (A and C) at position 80–81 in the reference sequence were changed to C and A in the subject, which changed the codon GAC to GCA (Figure 2). Similarly, in seq1 and seq3, the nucleotide base ‘A’ at position 144 was converted to G in the subject, which changed the codon sequence (GAA> GAG) (Figure 2). The variation in the nucleotide triplet from GAC to GCA (AC > CA allele) caused an amino acid change from aspartic acid to alanine, whereas the other variation of GAA to GAG (AA > AG allele) caused no change.

The detailed results of amino acid variations generated by the online BLASTx tool are shown in Table 7.

**Figure 3:**
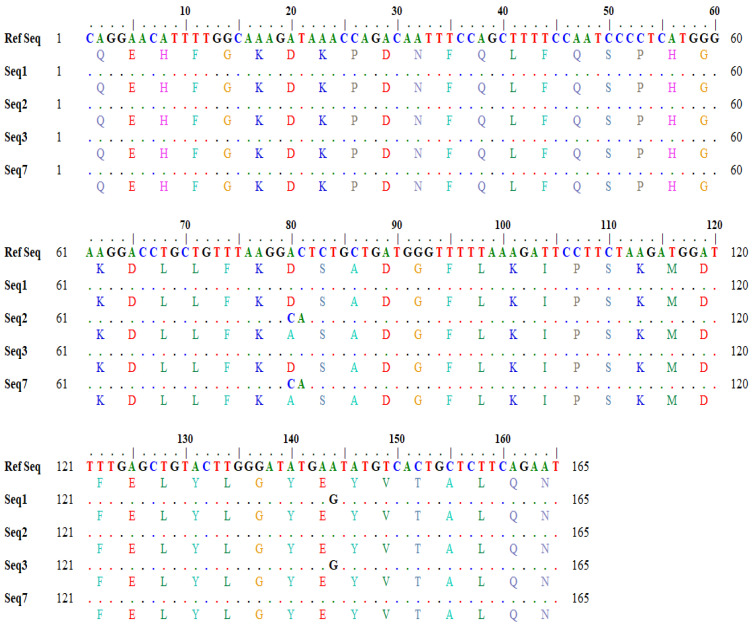
Detailed output of one-letter translation product showing amino acid variations.

Similarly, the output for a one-letter translational product showing variations in amino acids in the query and subject sequences was also generated through in silico tools (Figure 3).

#### 3.2.4. NCBI GenBank

The nucleotide sequences with G and CA alleles in which SNPs were found were submitted to the NCBI GenBank and were given accession nos. MZ892873 and MZ892874, respectively (Table 8).

### 3.3. Verification Tools for SNPs

#### 3.3.1. PROVEAN Scores

The PROVEAN tool identifies nonsynonymous variants that are functionally important by filtering variant sequences (Table 9). The PROVEAN analysis revealed that two SNPs were deleterious and one SNP was neutral. PROVEAN scores of ≤−2.5 reflected a damaging effect on an amino acid variant, whereas scores of >−2.5 indicated a neutral effect.

#### 3.3.2. PolyPhen-2

In this study, the variations at positions 320 and 321 (aspartic acid to alanine) were found to most likely be damaging, with a score of 0.996 (sensitivity: 0.36; specificity: 0.97). The mutation at position 341 was found to be benign, with the amino acid glutamic acid present in both the subject and query sequences. The variants classified as benign and damaging corresponded to numerical scores of 0 and 1.

#### 3.3.3. I-Mutant

In the present study, the I-Mutant tool was used to determine whether the SNPs found would increase or decrease the stability of the transferrin protein (Table 10). The findings showed that the SNPs (A > C, C > A) that caused an amino acid change from aspartic acid to alanine had a free-energy value <−0.5 kcal/mol, which indicated they were largely unstable, and there was a decreased stability of the protein. Similarly, the other transitional variation (A > G) was found to be neutral (≤0.5 kcal/mol), and there was no reported change in stability.

#### 3.3.4. MUpro

In this study, the results for mutations found at positions 320 (D > A) and 321 (D > A) showed that the change in the amino acid was found to decrease the protein stability, whereas no change in stability was found at position 341 (E > E).

#### 3.3.5. Sorting Intolerant from Tolerant (SIFT)

In this approach, scores for the amino acid residue were generated, and the values ranged from 0 to 1. The threshold intolerance values for SNPs were 0.05 or less; and for tolerance, the cutoff point was ≥0.05. In this study, the amino acid substitutions at positions 320 (Asp to Ala) and 321 (Asp to Ala) had a score of 0.01 and were found to affect function of the protein, whereas substitution at position 341 (Glu to Glu) had a cutoff value of 1.00 and did not affect the protein function, as the variation was tolerable.

The summary of the results validated by different computational tools has been presented in the tabular form (Table 11).

#### 3.3.6. Phylogenetic Analysis

**Figure 4:**
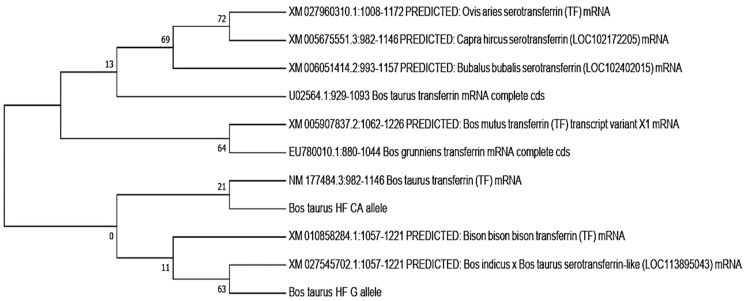
Evolutionary relationships of transferrin genes by using the neighbor-joining method.

In this study, the phylogenetic analysis revealed that the bovine transferrin CA allele was closely related to *Bos taurus* transferrin, while the *Bos taurus* G allele showed a close relation to the cross of the *Bos indicus* × *Bos taurus* serotransferrins, followed by the *Bison bison* transferrin. Both the CA and G alleles showed the least relation to *Capra hircus*, *Ovis aries*, and *Bubalus bubalis*. The neighbor-joining method was used to determine the evolutionary tree (Figure 4) [27]. MEGA X (version 2020) was used to evaluate evolutionary history, and the maximum composite likelihood method was used to determine evolutionary distances [28]

## 4. Discussion

Genetic polymorphism studies have extended our knowledge regarding to the immunity of the mammary gland, and has helped researchers globally to decipher mastitis resistance in some dairy cattle [29].

In this study, a 332 bp fragment of the bovine transferrin gene of the Holstein Friesian dairy breed of cattle was sequenced. Sequence homology of our fragment, when compared with the publicly available online databases, revealed 100% homology with the transferrin segment of *Bos taurus* and *Bison bison*. The same gene segment shared 99.45% homology with segments of *Bos indicus*, *Bubalus bubalis*, *Bos mutus*, *Bos grunniens*, and 98.90% with *Ovis aries* and *Capra hircus*. In our study, three novel SNPs: 14627A > C (exon 8), 14628C > A (exon 8), and 14691A > G (exon 8) were found in Holstein Friesian dairy cows by direct sequencing and compared with the reference sequence (NM_177484.3). The sequences of SNPs g.14627A > C, g.14628C > A, and g.14691A > G were submitted to the National Center for Biotechnology Information under accession nos. MZ892874 and MZ892873, respectively. Variations in two nucleotide DNA sequences at positions 230 (GAC > GCA) and 231 (GAC > GCA) were found to be nonsynonymous SNPs, and thus corresponded to an amino acid change (aspartic acid to alanine). Similarly, the variation at position 294 (GAA > GAG) was synonymous, and did not lead to an amino acid change (glutamic acid) in both the subject and query sequences. In this study, we found that the variation in the AC > CA allele caused an amino acid change that likely would affect the protein structure and function. Nonsynonymous SNPs that generate variants modify not only the protein’s tertiary structure, but also affect its function and stability, and also may form deleterious phenotypes [30]. The possible reasons for this are fourfold. First, the SNPs at positions g.14627A > C and g.14628C > A in exon 8 were nonsynonymous mutations (GAC (Asp) > (GCA) (Ala)) at the 230th and (GAC) (Asp) > (GCA) (Ala) 231st amino acids, in the vicinity of the C-terminal Fe^3+^ binding sites. Secondly, bacterial agents cause most cases of mastitis in dairy cows. In many species, including cattle, transferrin is a major glycoprotein that ensures transport of Fe^3+^ ions through biological fluids from sites of absorption/heme degradation to sites of storage and utilization [31]. For bacterial growth, sequestration of free iron is necessary, and this related to the antibacterial activity of transferrin. Third, the antimicrobial activity of transferrin is iron-independent, and is related to apo-Tf’s ability to decrease bacterial adhesion to surfaces [13]. Lastly, some variants of transferrin are linked to diseases.

Similar reports of polymorphisms were reported by the researchers in [32], who found three SNPs: g.1748G > A ss250608649, g.13942T > C ss250608650, and g.14037A > G ss250608651 in exon 8 of the transferrin gene. Similarly, in exon 12 of the TF cDNA sequence (accession no. U02564), an SNP (c.1455A > G) was found to induce substitution of Asp/Gly at the 469 peptide position [33]. In eight breeds of Spanish cattle and two groups of Holstein Friesian dairy animals, a potential association between milk fat yield and cSNP (c.1455A > G) was evaluated, and the results showed a significant association (*p* < 0.0006), with the G allele associated with high production of fat [12]. Within the two groups, a significant difference in genotypic frequency was also detected (*p* < 0.0028), which suggested that the Tf gene also effects milk fat production. Two SNPs, A14037G and C14081T, were revealed in 165 samples by direct sequencing and comparison with a reference sequence (NW_001493777) [34].

In our study, we used in silico tools to determine the effect of SNPs on protein structure and function. PROVEAN software was used to determine the impact of polymorphisms on the protein function and structure. The PROVEAN scores were evaluated based on homologs collected from NCBI datasets. The prediction scores had two types of threshold values: below −2.5 was considered deleterious, and above −2.5 was considered neutral. The findings of the present study revealed the Tf gene of *Bos taurus* to be highly polymorphic. In the present study, the amino acid substitution was found to be neutral and deleterious. The neutral substitution clearly indicated that the structure and function of protein was not impaired, and this will help in preventing susceptibility to disease and also gives hope to future selections, whereas the opposite results were associated with a deleterious substitution. In this study, since the substitution of amino acids revealed both deleterious and neutral effects, this indicated that in any efforts to increase the quantity of beneficial alleles, there was also a high tendency to increase the number of deleterious alleles.

Polyphen-2 analysis revealed that one SNP was benign, while two SNPs were most likely damaging. The mutation at position 341 (E > E) was benign; whereas at positions 320 (D > A) and 321 (D > A), the mutations were most likely damaging.

In the present study, the effects of nsSNPs were also analyzed through the I-Mutant tool, with results obtained in the form of mutant effects on the protein stability with a reliability index at pH 7.0 and temperature of 25 °C [35]. Glu341Glu showed no change in the Tf protein stability, whereas Asp320Ala and Asp321Ala were found to decrease the protein stability.

In the present study, the MUpro tool showed two mutations that changed amino acids and were found to decrease the protein stability, whereas the synonymous mutation showed no effect on stability. Furthermore, in this study, the SIFT tool was used with prediction scores assigned from 0 to 1, representing the normalized probability that the change in amino acid was tolerated. Generally, the SIFT tool is used to predict whether the substitution of an amino acid will affect protein function within correlated genes and domains over evolutionary times [36]. The SIFT results predicted that two amino acid substitutions at positions Asp320Ala and Asp321Ala would affect the function of proteins, with a deleterious tolerance index score of 0.01 for each. The substitution Glu341Glu was tolerated and did not affect the protein function. A similar study reported the use of three different in silico computational tools (PROVEAN, SIFT, and I-Mutant) [30]. The same study identified 14 variants as deleterious using the PROVEAN tool, while SIFT identified 15 variants that affected the protein function, and I-Mutant found 16 variants that were deleterious and were efficient in destabilizing proteins [30]. In another in silico study with an A122V substitution, the effect on the bovine CXCR1 protein was determined, and it was found to have a tolerable (SIFT), benign (PolyPhen-2) and nondeleterious (I-Mutant) effect [37].

Many studies have compared the effectiveness of these widely used pathogenic variant predictors [38,39]. Although each tool may outperform others in some aspect, some studies indicated that the accuracies of contemporary functional prediction tools were likely to be considerably lower than reported in their original publicized methods [38]. Several prediction tools provide different results because they utilize methods with different features; therefore, dissimilar outcomes would be expected at some point in time [40]. However, all the tools provided sufficient evidence of the deleterious effects of the Asp320Ala and Asp321Ala nsSNPs. Many nsSNPs can modify enzyme activity, disrupt protein interactions, and destabilize the structures of proteins [41], but it is not commonly known that some features of milk components in Holstein Friesian cows may potentially be altered by these nsSNPs. Therefore, in the native-type Tf protein, we predicted deleterious and drastic effects on the protein structure/function due to a mutation from Asp to Ala at residue positions 320 and 321. Unfortunately, because there were no previous bioinformatic data available on exon 8 SNPs in the bovine transferrin gene, we were not able to compare our data as such. Nonetheless, pilot data has been provided by this study that will filter nsSNP likelihood to determine whether these variations affect the function of the Tf protein. Further, deleterious nsSNPs have a profound effect on the structure and interaction of proteins, with likely impact on the quality of some milk components. Therefore, as such, these data provide the first evidence of the nature of the missense mutation in this portion of the bovine Tf gene. However, in other species, missense mutations are likely to effect the structure and function of transferrin protein. We believe that a combination of a DNA-sequencing-based method and various in silico tools (PROVEAN, I-Mutant, SIFT, MUpro, and Polyphen-2) could prove to be a useful tool for geneticists and breeders to identify unknown SNPs that could be useful for determining a possible impact on the structure and biological function of a protein.

In the present study, from about 500 replicates, a bootstrap consensus tree was inferred that was taken to signify the evolutionary history of the analyzed taxa [42,43]. The corresponding branches to partitions reproduced in fewer than 50% bootstrap replicates collapsed. In this analysis, 16 nucleotide sequences were involved. In each sequence pair, all the ambiguous positions were removed (pairwise deletion). In the final dataset, a total of 165 positions were present.

## 5. Conclusions

In the bovine transferrin gene, three SNPs—14627A > C (exon 8), 14628C > A (exon 8), and 14691A > G (exon 8)—were found by direct sequencing and compared with the reference sequence (NM_177484.3). The sequenced G and CA alleles were submitted to the NCBI GenBank and given accession nos. MZ892874 and MZ892873, respectively.

Phylogenetic analysis was conducted through MEGA X, which revealed the bovine transferrin CA allele was closely related to the *Bos taurus* transferrin, whereas the G allele was closely related to a cross of the *Bos indicus* × *Bos taurus* serotransferrins. Both alleles were least related to *Capra hircus*, *Ovis aries*, and *Bubalus bubalis*.

## Figures and Tables

**Table 1:** PCR product length and primers used, along with their melting temperature.

Target	Primer Seq.	Amplicon Size (bp)	Gene Region	T (°C)
Transferrin	Forward: 5′-TCTGACTGCCCTCTCTCCT-3′ Reverse: 5′-CTCCTAGAGCCACATGATCC-3′	332	14,432~14,764	57

**Table 2:** Concentrations and volumes of reagents used in PCR.

S. No	Reagents	Concentration	Volume
1	PCR master mix	(0.2 mm dnTP’s, 1.5 mm MgCl_2_ and 1 unit TaqDNA polymerase)	12.5 µL
2	Forward primer (fp)	10 pg/µL	1 µL
3	Reverse primer (rp)	10 pg/µL	1 µL
4	Template DNA	250 ng/µL	1 µL
5	Nuclease free water	variable	9.5 µL
Total			25 µL

**Table 3:** PCR cycle conditions.

Steps	Temperature	Time Interval
Initial denaturation	94 °C	5 min
I. Denaturation	94 °C	1 min
II. Annealing	56 °C	45 s
III. Extension	72 °C	45 s
Steps I–III repeated for 32 cycles		
Final extension	72 °C	5 min

**Table 4:** Query sequences producing significant alignments with the top 10 species.

Scientific Name	Max Score	Total Score	Query Cover	E Value	Per. Ident	Acc. Len	Accession
*Bison bison* TF	305	305	52%	1.00 × 10^−78^	100	2624	XM 010858284.1
*Bos taurus* TF mRNA	305	305	52%	1.00 × 10^−78^	100	2584	NM 177484.3
*Bos taurus* TF mRNA cds	305	305	52%	1.00 × 10^−78^	100	2338	U02564.1
*Bos indicus* × *Bos taurus*	300	300	52%	5.00 × 10^−77^	99.39	4276	XM 027545702.1
*Bubalus bubalis*	300	300	52%	5.00 × 10^−77^	99.39	2557	XM 006051414.2
*Bos mutus*	300	300	52%	5.00 × 10^−77^	99.39	2463	XM 005907837.2
*Bos mutus*	300	300	52%	5.00 × 10^−77^	99.39	2115	NM 001311351.1
*Bos grunniens*	300	300	52%	5.00 × 10^−77^	99.39	2115	EU 780010.1
*Ovis aries*	294	294	52%	2.00 × 10^−75^	98.79	2579	XM 027960310.1
*Capra hircus*	294	294	52%	2.00 × 10^−75^	98.79	2552	XM 005675551.3

**Table 5:** SNPs identified and types of mutations in bovine transferrin gene.

S. No.	SNP and Its Position	Nucleotide		Type of Variation
		Reference	Crossbred HF (Genotype)	
		(PPR1) Acc. No.: NM_17744.3, *Bos taurus*.		
1	−230 bp A > C	A	C	Transversion
2	−231 bp C > A	C	A	Transversion
3	−294 bp A > G	A	G	Transition

**Table 6:** NovelSNPer detailed output file with transcript variation per line.

Name	Chr	Start	End	Allele	Refallele	AA	Ref AA	Type	SNP
var1	1	230	230	A	C	Aspartic acid	Alanine	SNP	Novel
var2	1	231	231	C	A	Aspartic acid	Alanine	SNP	Novel
var3	1	294	294	A	G	Glutamic acid	Glutamic acid	-	-

**Table 7:** Amino acid variation in subject and query protein sequences.

S. No.	Position	Amino Acid	
		Subject	Query
1	320	D (Asp)	A (Ala)
2	321	D (Asp)	A (Ala)
3	341	E (Glu)	E (Glu)

**Table 8:** Submission of nucleotide sequences with SNPs to the NCBI GenBank.

SNP	Codon	Allele	Accession No.
A > C, C > A	GAC > GCA	CA	MZ892873
A > G	GAA > GAG	G	MZ892874

**Table 9:** Prediction and scores generated by the PROVEAN tool.

Variant	PROVEAN Score	Prediction (Cutoff = 2.5)
D320A	−6.879	Deleterious
D321A	−6.879	Deleterious
E341E	0.000	Neutral

**Table 10:** Protein stability reports generated by I-Mutant.

Position	Wild-Type Amino Acid	New Amino Acid after Mutation	Stability	Reliability Index	pH	Temperature
320	D	A	Decrease	7	7.0	25 °C
321	D	A	Decrease	7	7.0	25 °C
341	E	E	No change	3	7.0	25 °C

**Table 11:** Validation of results obtained from different bioinformatical tools.

Mutation	Provean	PolyPhen-2	I-mutant	Mupro	SIFT
A230C	Deleterious	Probably damaging	Decreased	Decreased stability	Affected protein function
C231A	Deleterious	Probably damaging	Decreased	Decreased stability	Affected protein function
A294G	Neutral	Benign	No change	Did not affect stability	Did not affect protein function

## Data Availability

All the data generated in this study were published in the article.
